# Advances in the Development of a Universal Influenza Vaccine

**DOI:** 10.3390/antib15040075

**Published:** 2026-08-11

**Authors:** Preanka Mudaly, Max Lee, Aishwarya Bhatta, Craig Thompson

**Affiliations:** Warwick Medical School, University of Warwick, Coventry CV4 7AL, UK

**Keywords:** universal influenza vaccine, conserved epitope, antigenic drift, antigenic shift, immunogenicity, cross-reactive antibody, HA stalk, broadly neutralising antibody, nanoparticle vaccine, neuraminidase, T-cell immunity, mucosal immunity, correlates of protection, mRNA vaccine, immune imprinting, pandemic preparedness

## Abstract

Influenza remains a persistent global health threat, accounting for an estimated one billion infections and 350,000–600,000 deaths worldwide annually. Beyond substantial mortality and morbidity, influenza imposes major socio-economic costs through healthcare utilisation and productivity losses, amounting to tens of billions of dollars each year. Current seasonal vaccination strategies are constrained by rapid antigenic evolution, which necessitates frequent vaccine reformulation and contributes to suboptimal vaccine efficacy across seasons. These drawbacks highlight the need for a universal influenza vaccine (UIV) that protects against all seasonal and potential pandemic influenza strains via a single dose. This review evaluates the feasibility of a UIV as an alternative to a seasonal influenza vaccine (SIV) by consolidating evidence from current UIV candidates in preclinical and clinical development across a diverse range of platforms and target epitopes. Preclinical studies and clinical trials have demonstrated the safety and tolerability of multiple candidates, as well as their ability to elicit broadly cross-reactive immune responses. However, several challenges remain regarding the long-term efficacy of these vaccines. In particular, pre-existing host immunity can shape subsequent vaccine responses, with immune imprinting introducing biases toward previously encountered influenza strains, potentially reducing vaccine effectiveness. In addition, the genetic plasticity of influenza viruses, driven by ongoing mutations and genetic reassortment, raises concerns about the ability of a single vaccine formulation to maintain broad and durable protection. Overall, several UIV candidates have shown promising early results. Although ecological and immunological barriers remain, a UIV could become a viable alternative to, or eventual replacement, for SIVs.

## 1. Introduction

Influenza viruses are enveloped, negative-sense, single-stranded RNA viruses belonging to the Orthomyxoviridae family [1,2]. Of the four types (A, B, C and D), influenza A poses the greatest threat to human health, owing to its capacity to undergo both antigenic drift and antigenic shift. Antigenic drift is driven by the error-prone nature of the RNA-dependent RNA polymerase, which introduces point mutations across the viral genome during replication, while antigenic shift arises from genetic reassortment among the eight RNA gene segments during co-infection of a single host cell [3,4,5]. Two major surface glycoproteins, haemagglutinin (HA) and neuraminidase (NA), mediate host-cell entry/attachment and viral egress, respectively, and serve as the basis for the classification and nomenclature of influenza A subtypes and the lineage structure of influenza B viruses [6,7,8,9].

Influenza has caused multiple pandemics throughout history, many of which originated from the zoonotic spillover of influenza A viruses from animal reservoirs into human populations [10,11,12]. Notable examples from the past century include the 1918 Spanish flu (H1N1), the 1957 Asian flu (H2N2), the 1968 Hong Kong flu (H3N2) and the 2009 influenza A swine flu pandemic (H1N1pdm09) [13,14,15].

Current influenza management strategies rely on SIVs, antiviral therapies and non-pharmaceutical interventions [11,16,17,18,19,20]. SIVs are designed to induce antibody responses against the immunodominant head domain of HA and are reformulated annually based on World Health Organisation (WHO) global surveillance data to match predicted circulating strains approximately six months ahead of each influenza season [11]. However, vaccine effectiveness remains variable, typically ranging from 10-60%, with the lower end reflecting seasons of poor antigenic matching between the vaccine and circulating strains [4,17,21]. Protective immunity following seasonal vaccination also wanes over time, necessitating repeated annual vaccination to maintain protection [11,14].

There are several further limitations of current SIVs. Widespread reliance on egg- based vaccine production is a lengthy process and may introduce adaptive mutations that alter antigenicity and compromise vaccine performance [7,11]. Dependence on conventional egg-based manufacturing also limits production flexibility and rapid scalability in response to emerging influenza threats [7,11]. Additionally, SIV-induced immunity is predominantly strain-specific, providing limited cross-reactivity against antigenically distinct strains and little protection against emerging pandemic viruses [11,14].

Variability of the virus is driven largely by antigenic drift: the gradual accumulation of amino acid substitutions at key antigenic sites in surface glycoproteins, particularly HA [1,3,4]. Even modest changes can substantially alter viral antigenicity, enabling circulating strains to evade pre-existing antibody responses [4,22]. Importantly, antigenic drift does not occur uniformly across the globe; phylogenetic and antigenic mapping studies have identified tropical regions that act as reservoirs for emerging, drifted influenza variants, which subsequently disseminate globally and complicate strain prediction [4,5,23].

A related concept is antigenic thrift, which proposes that the antigenic evolution of influenza is not unbounded but, constrained to a limited repertoire of antigenic variants that recycle over decadal timescales [24,25]. This model has direct implications for broadly protective vaccine design, as it suggests that the antigenic space requiring coverage may be narrower than the high mutation rate of influenza would otherwise imply. The implications of antigenic thrift for UIV target selection are discussed further in Section 3.5.

**Figure 1:**
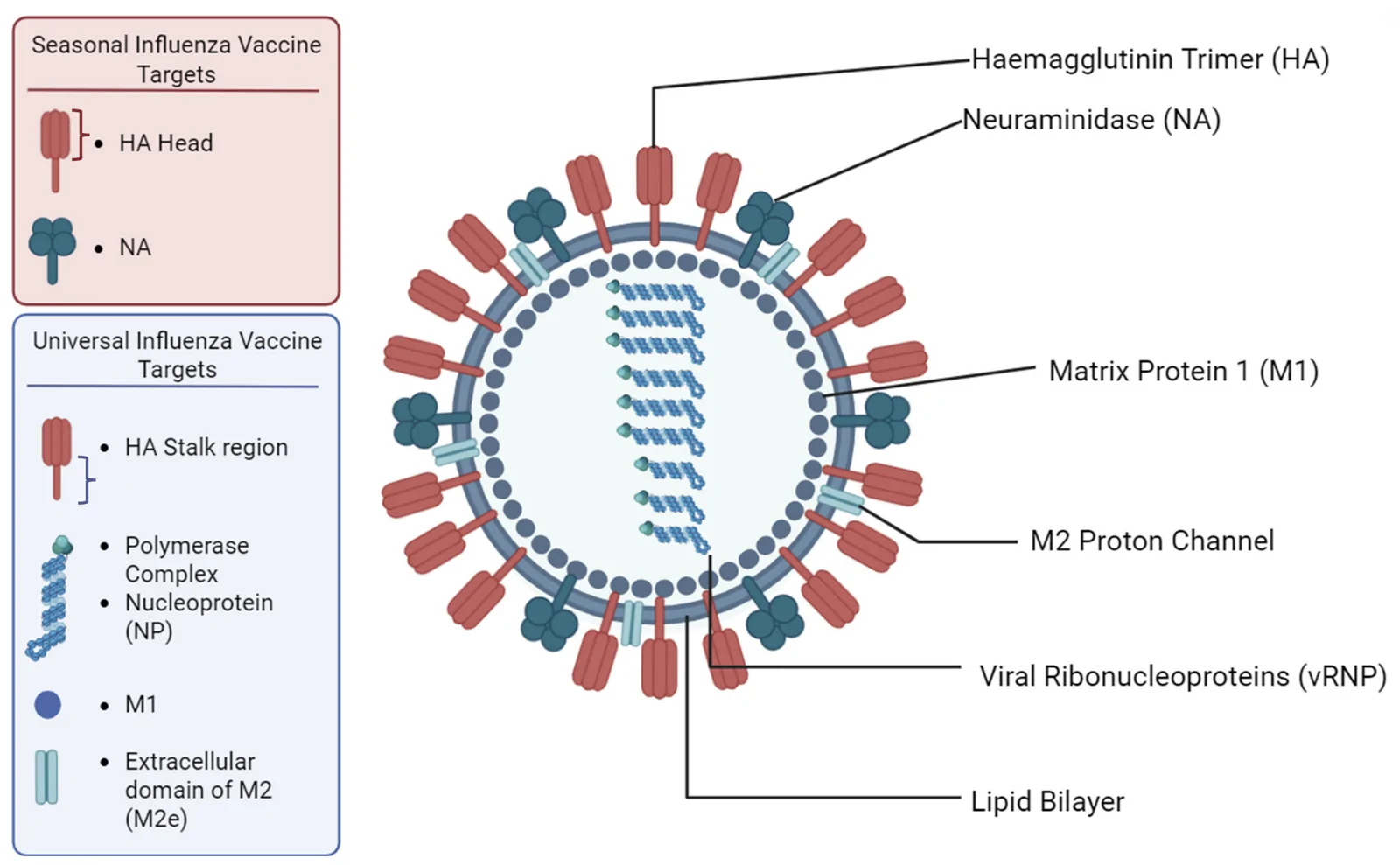
Schematic representation of structural components of influenza virus, illustrating the target regions of UIV and SIV candidates. Surface glycoproteins (HA and NA) and internal proteins (NP, M1 and M2e) are indicated alongside their respective vaccine targeting strategies.

A UIV aims to overcome these limitations by targeting conserved viral components—principally, the HA stalk domain [7,11,26,27,28,29,30,31,32,33], the extracellular domain of M2 (M2e), internal proteins including the nucleoprotein (NP) and matrix protein (M1) [34,35,36,37,38,39,40,41,42,43]—to elicit broad, durable and cross-reactive immunity independent of strain-specific antigen matching. The conserved viral targets exploited by UIVs, compared with those of SIVs, are illustrated in Figure 1.

The National Institute of Allergy and Infectious Diseases (NIAID) has defined strategic benchmarks for a UIV: protection against both group 1 and group 2 influenza A viruses, at least 75% efficacy against symptomatic infection, a minimum duration of protection of one year, and suitability for all age groups [14,44].

UIV candidates currently span a diverse range of platforms and remain largely in preclinical or early-phase clinical development [45,46]. A significant challenge in evaluating these candidates is the lack of validated correlates of broad protection. Haemagglutinin inhibition (HAI) titres serve as the established benchmark for SIV efficacy, but they measure antibodies directed against the HA head—precisely the target that most UIV strategies are designed to move away from. Candidates eliciting stalk-directed antibodies, anti-NP immunoglobulin (IgG) or CD4+/CD8+ T-cell responses may demonstrate a negligible HAI titre despite generating functionally protective immunity [23,47,48,49,50,51]. Establishing standardised immune endpoints that capture these non-classical responses is therefore critical for the clinical advancement of UIV candidates.

This review evaluates current progress in UIV development, examining candidate platforms, targeted epitopes, the breadth of elicited cross-reactive immune responses, and the projected global health impact of implementation.

## 2. Current Universal Influenza Vaccine Candidates

**Figure 2:**
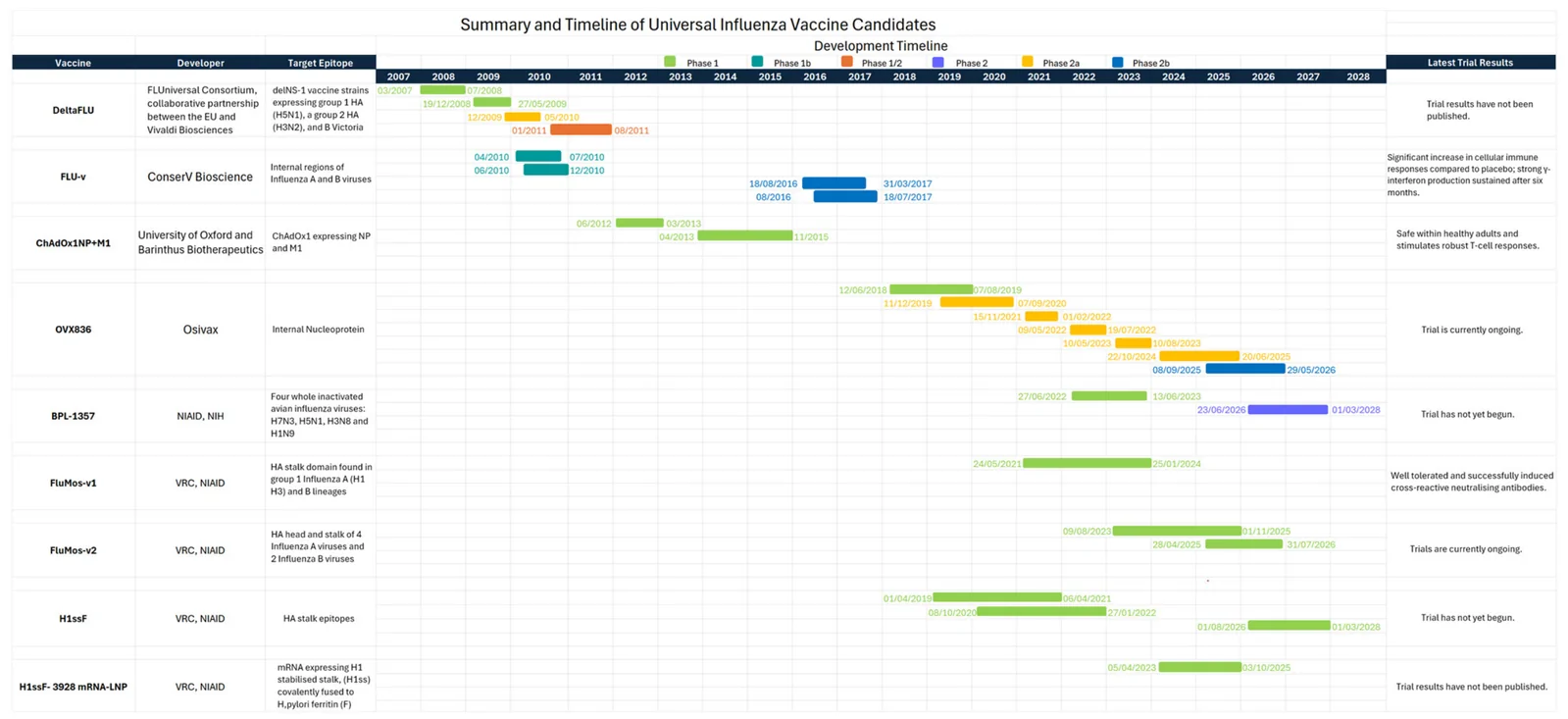
Timeline of clinical trial phases. Clinical trial progression for the leading UIV candidates illustrates the duration and phase of clinical trials across the years, highlighting those in phase 1 and those that have advanced to phase 2 [56,57,58,59,60,61,62,63,64,65,66,67,68,69,70,71,72,73,74,75,76,77,78,79,80,81,82].

Multiple UIV candidates have now advanced into clinical development across a range of platforms, including recombinant protein subunit, peptide-based, self-assembling nanoparticles, viral-vectored, nucleic acid (DNA and mRNA-LNP), whole-virus-inactivated, and intranasal live-attenuated vaccines [2,36,52,53,54,55]. Figure 2 summarises the leading candidates, their developing companies, their targeted epitopes, and current clinical trial status, along with the clinical progression timeline (dates estimated where trials are ongoing or not yet completed) and latest results, where published. Table 1 provides a summary of immune endpoints of the discussed UIV candidates.

### 2.1. Recombinant Protein-Based Vaccines

Recombinant protein-based platforms express specific viral immunogens in heterologous systems to produce purified, non-infectious subunit vaccines. Several UIV candidates use this approach to target conserved internal or surface-proximal antigens [82,83,84,85,86].

FLU-v, developed by ConserV Bioscience (formerly PepTcell Limited), is a multicomponent synthetic peptide vaccine targeting conserved epitopes within M1, M2, NP-A, and NP-B of both influenza A and B viruses. In its phase 2b randomised, double-blind, placebo-controlled trial (NCT02962908), vaccination produced significant increases in IFNγ-and granzyme-B-secreting cells by day 42, consistent with robust CD4+ and CD8+ T-cell activation and cytolytic potential [62,63]. Notably, T-cell responses showed strong cross-reactivity against diverse influenza A subtypes (H1N1, H3N2, H5N1 and H7N9), though cross-reactivity to influenza B was more limited, likely reflecting greater sequence divergence in the targeted epitopes between influenza A and B viruses [35,87,88].

The G1mHA vaccine (Janssen Vaccines and Prevention) takes a structure-based approach, presenting a stabilised trimeric group 1 influenza A HA stalk immunogen designed to focus antibody responses on the conserved stalk domain rather than the immunodominant and variable head. Preclinical data from non-human primate studies showed that adjuvanted formulations induced durable stalk-directed antibody responses with cross-group 1 neutralisation breadth [89,90]. The phase 1/2a randomised, double-blind, controlled trials (NCT05901636) enrolled 170 healthy adults aged 18–45. Participants received one or two doses of 45 µg or 135 µg of G1mHA, with or without aluminium hydroxide adjuvant on days 1 and 57 [89]. Both doses demonstrated an acceptable safety profile with no vaccine-related serious adverse events. Binding antibody responses to H5 HA showed 6.5–16.4-fold increases at day 29, compared to a 2.1–2.2-fold increase for SIV, with 64–100% of participants showing a greater than 4-fold increase, while the SIV only achieved an increase in 14–17% of participants. Neutralising antibody responses demonstrated 7.9–15-fold increases versus a 1.9-fold increase for SIV, while antibody-dependent cellular cytotoxicity (ADCC) responses ranged from a 4.9–8.6-fold increase compared to a 2.2-fold increase for SIV [89,90].

Recombinant protein-based platforms typically rely on adjuvants to achieve adequate immunogenicity, for example, the FLU-v vaccine uses Montanide ISA-51. This increases the complexity of formulations, as well as potential costs [83,84]. The antigen selection inherent to recombinant protein-based vaccines limits sequence divergence between targeted epitopes and circulating viral variants; for example, FLU-v’s strong cross-reactivity against diverse influenza A subtypes is substantially weaker against influenza B due to greater sequence divergence in internal protein epitopes [35,87]. Additionally, immunological responses have a shorter duration compared to whole-virus approaches and this could potentially necessitate booster doses, which would not align with the goals of a UIV in terms of providing long-lasting durability from one dose.

### 2.2. Nanoparticle-Based Vaccines

Nanoparticle-based vaccines present multiple copies of an antigen in highly ordered, repetitive arrays that mimic viral surface geometry. This multivalent display crosslinks B-cell receptors, enhancing germinal-centre responses and enabling the recruitment of B cells that recognise conserved epitopes, thereby broadening the antibody response beyond immunodominant, strain-specific determinants [30,85,91,92,93].

FluMos-v1 is a self-assembling mosaic nanoparticle vaccine developed by the Vaccine Research Centre (VRC) at NIAID. It displays HA from an H1N1 strain, an H3N2 strain, a B/Victoria lineage strain and a B/Yamagata lineage strain, using a ferritin nanoparticle structure designed to focus B-cell responses on shared epitopes across displayed variants [94,95]. In its phase 1, open-label, dose-escalation trial (NCT04896086), FluMos-v1 was administered to healthy adults aged 18–50 years. The vaccine was found to be safe and well tolerated and induced both strain-specific and cross-reactive neutralising antibodies targeting the HA stalk region [67]. Critically, the mosaic design elicited stalk-directed antibody responses, even in participants with pre-existing head-directed immunity, suggesting that multivalent nanoparticle presentation can partially overcome the immunodominance hierarchy that typically limits stalk antibody induction [67,96,97]. FluMos-v2, currently in phase 1 trials (NCT05968989 and NCT06863142), extends this platform to a hexavalent formulation incorporating six HA antigens to broaden strain coverage further.

H1ssF is a headless HA stalk-focused ferritin nanoparticle vaccine, also developed at VRC-NIAID, that exclusively presents the stabilised group 1 HA stalk domain to eliminate competing head-directed responses. In a first-in-human phase 1 dose-escalation trial (NCT03814720), H1ssF was found to be safe and induced cross-group 1 neutralising antibodies with breadth across H1, H5, H6, and H9 subtypes in healthy adults aged 18–70 [45]. Notably, these neutralising antibody responses were durable, persisting for over one year post vaccination, providing proof of concept that engineered nanoparticle immunogens can elicit broadly neutralising, stalk-directed antibodies in humans by presenting the HA stalk, despite the pre-existing immunological bias towards head-directed responses [45].

OVX836, developed by Osivax, takes a mechanistically distinct approach within this platform category. Rather than targeting surface glycoproteins, OVX836 uses a proprietary technology in which the influenza A nucleoprotein is genetically fused to the OVX313 heptameric oligomerisation domain, causing NP to self-assemble into a stable homo-heptameric nanoparticle displaying seven full-length copies of the antigen. This architecture markedly enhances NP immunogenicity relative to monomeric or trimeric NP formulations. OVX836 elicits polyfunctional CD4+ T cells co-producing IFNγ, IL-2, and TNFα, with CD8+ T-cell responses emerging at doses ≥ 300 μg [79]. The vaccine also induces anti-NP IgG responses detectable from days 8–29 post vaccination, with a profile consistent with potential ADCC activity [98]. OVX836 has demonstrated safety across phase 1 (NCT03594890) and multiple phase 2 trials (the latest, NCT05569239, enrolled 2850 participants to assess efficacy, safety and immunogenicity), with T-cell responses showing cross-reactivity across influenza A strains [51,88,98,99,100,101].

Nanoparticle-based platforms primarily face challenges in manufacturing and scalability across diverse global regions. These vaccines require precise engineering to generate uniform, self-assembling, multivalent structures [91,92,93]. Manufacturing costs for nanoparticle vaccines typically exceed those of conventional inactivated vaccines; therefore, for regions that do not already have the necessary manufacturing sites in place, this could be a costly technological hurdle to overcome [91]. In addition to this, nanoparticle vaccines have strict cold-chain requirements due to their limited thermal durability, which can make storage and transport difficult, limiting the overall accessibility of the vaccine [96,97].

### 2.3. Viral-Vectored Based Vaccines

Viral vector-based UIV candidates use replication-deficient viruses to deliver influenza antigens and induce both humoral and cellular immunity. Adenoviral vectors are particularly well suited to UIV development due to their capacity to elicit strong CD8+ T-cell responses and durable immunity following a single dose [28,36]. The ChAdOx1 platform has been validated at global manufacturing scale through the Oxford–AstraZeneca COVID-19 vaccine, providing a clinically and logistically proven pathway for large-scale deployment.

ChAdOx1NP+M1, developed by the University of Oxford in collaboration with Barinthus Biotherapeutics, uses the chimpanzee adenovirus Oxford 1 vector to co-express the conserved internal NP and M1 antigens. In a first-in-human phase 1 trial (NCT01818362), the vaccine was found to be safe and well tolerated, inducing polyfunctional CD4+ and CD8+ T cells producing IFNγ and TNFα, with antigen-specific T-cell responses maintained at 18 months post vaccination [102,103]. By targeting internal proteins that are highly conserved across influenza A subtypes and largely inaccessible to pre-existing strain-specific antibodies, this approach aims to provide heterosubtypic protection through T-cell-mediated clearance of infected cells rather than antibody-mediated neutralisation. This mechanism is complementary to the stalk-directed antibody strategies described in the preceding sections. However, clinical development of this candidate has not been advanced beyond chronic infectious diseases and autoimmunity; further clinical evaluation would require new sponsorship or academic partnership [103].

Pre-existing immunity to the vector in a substantial proportion of the global population, particularly for adenoviral vectors, constrains vaccine efficacy [28,36]. When targeting conserved internal proteins, as shown in ChAdOx1NP+M1, the absence of strain-specific neutralising antibodies may limit protection against infection. Moreover, vector-specific immunity elicited by primary vaccination may compromise the effectiveness of subsequent doses, as immune responses targeting the vector may limit antigen delivery and presentation upon re-administration [28,36,102,103].

### 2.4. Nucleic Acid Vaccines

Nucleic acid platforms, particularly mRNA, offer distinct advantages for UIV development: rapid iterative antigen design, the ability to encode structurally complex immunogens for in vivo expression, and proven scalable manufacturing validated at pandemic speed during the SARS-CoV-2 response [2,96,104,105]. Preclinical studies have demonstrated that multivalent mRNA constructs encoding conserved HA stalk and internal protein antigens can induce both broadly cross-reactive antibody and T-cell responses [104,105,106].

H1ssF-3928 mRNA-LNP, developed under the Collaborative Influenza Vaccine Innovation Centres (CIVICs) programme at VRC-NIAID, encodes the same stabilised H1 stalk–ferritin immunogen as the H1ssF-3928 nanoparticle vaccine (Section 2.2) but delivers it as an mRNA–lipid nanoparticle formulation for in vivo antigen expression. This approach is designed to combine the immunological benefits of the stalk-nanoparticle construct with the manufacturing speed and scalability of the mRNA platform. A phase 1 trial (NCT05755620) evaluating safety and immunogenicity in 50 healthy adults aged 18–49 years (inclusive) at three dosage levels (10 mcg, 25 mcg and 50 mcg) was completed, though results have not yet been published [66]. This trial is notable as the first UIV candidate evaluated under the CIVICs programme and will provide the first direct, head-to-head immunological comparison between a stalk-targeting mRNA vaccine and a conventional seasonal vaccine in humans. Two key questions remain for mRNA-based UIV candidates: first, whether the durability of the immune response is comparable to that of protein-based delivery, and second, whether cold-chain requirements (currently 2–8 °C for most LNP formulations) can be sufficiently reduced for global deployment, which is particularly important for low-income developing countries, where the burden of influenza is disproportionately high [66,106].

### 2.5. Whole-Virus Inactivated Vaccines

Whole-virus inactivated vaccines present the complete complement of viral surface and internal antigens, potentially engaging both humoral and cellular immunity against multiple conserved targets without requiring the antigen selection inherent to subunit and nanoparticle approaches [28,36].

BPL-1357, developed at NIAID/NIH, is a beta-propiolactone-inactivated whole-virus candidate containing four low-pathogenicity avian influenza strains: H7N3, H5N1, H3N8, and H1N9. The use of avian subtypes to which the human population is largely immunologically naïve is a deliberate design strategy intended to prime broad immune responses without the confounding effects of pre-existing head-directed immunity from prior seasonal influenza exposure. Its phase 1 randomised, placebo-controlled trial (NCT05027932) was conducted on 45 healthy adults aged 18–55 years, with the vaccine administered intramuscularly (IM) or intranasally (IN) in two doses 28 days apart. BPL-1357 demonstrated an acceptable safety profile and induced strain-specific and cross-reactive haemagglutinin-directed antibodies, including broadly neutralising responses extending beyond the four vaccine strains [107]. Early stability data suggest the vaccine may tolerate storage outside standard cold-chain conditions, which would be a significant advantage for deployment in low-resource settings [108]. A phase 2 human challenge trial (NCT07215858) evaluating protective efficacy against H1N1 challenge via both IM and IN routes has an estimated start date of 26 February 2026.

Whole-virus inactivated vaccine platforms could potentially face challenges with antigenic presentation and viral evolution. Although BPL-1357 contains low-pathogenicity avian influenza strains to which the population has minimal pre-existing immunity, this does not fully overcome the effects of immune imprinting or seasonal vaccinations [68]. Furthermore, whole-virus vaccines have strict cold-chain requirements, such as continuous temperature monitoring and sensitivity to light, limiting deployment in tropical regions—something that seasonal vaccines currently face [107,109].

### 2.6. Intranasal Live-Attenuated Vaccines

All previously described candidates are administered parenterally and primarily induce systemic humoral and cellular immunity. Intranasal live-attenuated vaccines offer a fundamentally different immunological approach by eliciting mucosal immunity at the respiratory epithelium—the primary site of influenza infection—including secretory IgA and tissue-resident memory T cells that can intercept the virus before systemic infection is established [28,36].

DeltaFLU, developed by the FLUniversal consortium (an EU-funded public–private partnership; Vivaldi Biosciences), is a live-attenuated intranasal vaccine containing replication-deficient influenza A and B strains engineered with deletion of the NS1 gene. NS1 normally functions as a virulence factor by antagonising the host type I interferon response; its removal renders the virus replication-deficient and prevents vaccine shedding while simultaneously enhancing interferon induction, effectively creating a self-adjuvating immunogen that eliminates the need for additional adjuvants [110]. This response, in turn, enhances T- and B-cell activity, inducing memory T- and B-cells and directing B-cell responses toward the conserved HA stalk domain, which is typically poorly immunogenic in conventional vaccination contexts. Preclinical evaluation in ferret and hamster models was carried out to assess immunogenicity, shedding, and biodistribution. Following this, a randomised, double-blind, placebo-controlled, phase 1 dose-escalation study was carried out in 48 healthy volunteers at five dose levels, with eight participants per dose level; results have not yet been published [56,110].

As an intranasal formulation, DeltaFLU offers practical advantages for global deployment beyond its immunological profile: needle-free administration may increase uptake, particularly in needle-averse populations and resource-limited settings where injection infrastructure is constrained, while the Vero cell-based manufacturing platform used by Vivaldi Biosciences offers a scalable, egg-independent production pathway with a production timeline of approximately seven weeks, which is considerably shorter than conventional egg-based vaccine manufacturing timelines [110].

Live-attenuated intranasal platforms must exhibit durable and stable attenuation, particularly for longer deployment distances, as although very rare, there is a risk of reversion to virulence [110]. In addition, for vaccines such as DeltaFLU, there is inadequate understanding of mucosal secretory IgA responses over parenteral approaches [28,36].

## 3. Discussion

### 3.1. Immunity and Protection

The candidates reviewed here demonstrate that multiple immunological pathways to broad influenza protection are under active clinical investigation. Stalk-directed nanoparticle vaccines (FluMos-v1 and H1ssF) have induced cross-group 1 neutralising antibodies in humans, with FluMos-v1 additionally eliciting stalk-directed responses in participants with pre-existing head-directed immunity [45,94]. Importantly, these stalk-directed antibodies are not captured by conventional HAI assays, which measure head-binding antibodies that block receptor attachment; instead, stalk antibodies mediate protection principally through Fc-dependent effector mechanisms, including ADCC and inhibition of viral membrane fusion [23,47,48,49,50,51]. This mechanistic distinction underscores the inadequacy of HAI as a universal correlate of protection for UIV candidates and reinforces the need for novel immune correlates, as discussed in the introduction.

In parallel, T-cell-targeting candidates have reached phase 2 evaluation. OVX836 and FLU-v both induce polyfunctional CD4+ and CD8+ T-cell responses against conserved internal viral proteins, with cross-reactivity across influenza A subtypes [35,87,88,98,99,100,101]. The ChAdOx1NP+M1 viral vector similarly generates durable NP- and M1-specific T-cell immunity, which is maintained 18 months post vaccination [102,103]. Across all platforms, phase 1 and 2 data consistently report acceptable safety profiles with low reactogenicity [45,100,102].

However, these results must be interpreted with caution. Efficacy against clinical endpoints remains undemonstrated for all reviewed candidates, and the durability of vaccine-induced cross-reactive responses beyond 12–18 months is largely unknown. Whether the antibody and T-cell responses observed in controlled trial settings translate to population-level protection against the full breadth of circulating strains is the critical unanswered question.

### 3.2. Comparing Antibody Responses Across Platforms

Different vaccine-platform architectures engage distinct components of the humoral immune response, with important implications for the breadth and durability of protection.

Nanoparticle-based platforms, in particular, have a strong ability to elicit HA stalk-directed IgG responses by promoting recognition of conserved epitopes and mitigating immunodominance associated with prior HA head-specific immune imprinting [1,7,28]. FluMos-v1 has been shown to induce stalk-directed antibodies, even in individuals with pre-existing HA head-specific immunity, as the display of diverse HA stalk epitopes largely increases the breadth of provided protection [45,111]. In contrast to this, recombinant protein-based platforms such as FLU-v pursue a complementary strategy by targeting internal viral proteins, including the NP and matrix proteins, which induce IgG1 and IgG3 antibodies, mediating ADCC rather than direct virus neutralisation [34,35,51]. Immune responses were maintained for at least six months post vaccination, representing a promising starting point for long-term durability; however, to meet NIAID criteria, protection would need to last at least one year [87].

Whole-virus inactivated platforms such as BPL-1357 continue to use the conventional HAI titres as their primary correlate of protection, which is particularly useful for interpretation of clinical data, as it is a standardised benchmark [23,39,111,112,113]. However, HAI titres primarily reflect strain-specific immunity and may underestimate broader protective mechanisms. Consequently, comparison of immunogenicity across platforms requires careful consideration of the distinct immune endpoints being measured. Table 1 summarises the range of primary endpoints used to assess UIV candidates across platforms.

Stalk-directed IgG responses induced by nanoparticle and whole-virus approaches are strongly associated with virus neutralisation and long-term immunity [7,28,114]. Recombinant protein-based UIVs successfully induce IgG1 and IgG3 subclasses, which mediate Fc-dependent effector functions, including ADCC and phagocytosis [32]. These mechanisms may contribute to cross-strain protection, even when there is limited neutralising activity. Antibody-mediated protection is influenced by the injection site [35,51,87]. Parenteral vaccination predominantly induces serum IgG, whereas intranasal platforms generate mucosal secretory IgA (sIgA), as demonstrated with the DeltaFLU vaccine [28,36,110]. sIgA is highly effective at neutralising viruses at the site of entry and may therefore provide better protection against viral replication and transmission. In contrast, IgG generally acts after infection has been established, reducing disease severity rather than preventing infection. Collectively, this can influence UIV development, motivating the aim to induce a range of antibody responses that balances the benefits of both IgG- and IgA-mediated immunity [28,36,96,110].

#### 3.2.1. Correlates of Protection and Assay Standardisation

A fundamental challenge in comparing UIV candidates across platforms is the absence of validated correlates of broadly protective immunity. The HAI titre of 1:40 remains the only regulatorily accepted immune correlate for influenza vaccination, yet this metric measures antibodies directed against the HA head domain, which is precisely the region that most UIV candidates aim to circumvent [7,28,114]. Stalk-directed antibodies, anti-neuraminidase responses, Fc-dependent effector functions (ADCC and ADCP), mucosal sIgA, and T-cell-mediated immunity each contribute to protection through distinct mechanisms, but none has an established quantitative threshold equivalent to HAI 1:40 [28,36,115]. Consequently, candidates evaluated by different assays cannot be meaningfully ranked against one another. The problem is compounded by methodological heterogeneity: pseudovirus neutralisation, microneutralisation, enzyme-linked lectin assay (ELLA), ELISpot, and intracellular cytokine staining each captures different facets of the immune response, and standardised protocols across laboratories remain limited [115,116].

Rather than relying on a single immune correlate similar to the HAI titre, future evaluation of UIVs will likely require a standardised panel of immune endpoints that integrate multiple complementary measures of protection. Depending on the vaccine platform, these may include stalk-directed neutralising antibodies, neuraminidase inhibition titres, Fc-mediated effector functions such as ADCC, mucosal secretory IgA responses and antigen-specific CD4+ and CD8+ T-cell immunity [28,36,115,116]. Prospective clinical studies correlating these immune responses with sufficient protection against laboratory-confirmed influenza infection will be essential to determining and predicting vaccine efficacy [115,116].

Establishing validated, broadly accepted immune correlates and harmonised assay panels should be considered a priority for the field, as their absence currently represents a bottleneck not only for candidate comparison but also for regulatory licensure of UIV candidates intended to protect through non-traditional immune mechanisms.

#### 3.2.2. Neuraminidase as a Complementary Vaccine Target

Although the majority of UIV candidates discussed in this review target the HA stalk, internal proteins, or whole-virus preparations, NA represents an underexploited but immunologically significant target for broadly protective influenza vaccination. Anti-NA antibodies do not prevent viral attachment or entry but restrict virion release from infected cells and limit viral spread within the respiratory tract, providing a mechanistically independent layer of protection that complements HA-directed immunity [50,117]. Epidemiological and clinical evidence indicates that anti-NA immunity, measured by ELLA, is an independent correlate of reduced viral shedding, symptom severity, and duration of illness and that NA inhibition (NAI) titres predict protection independently of HAI titres [117,118]. Despite this, NA has historically received less attention in vaccine design, partly because NA content is neither standardised nor consistently measured in current seasonal vaccines [118]. Recent efforts to develop NA-based or NA-inclusive vaccine candidates, including recombinant NA proteins and bivalent HA-stalk/NA formulations, suggest that co-targeting both surface glycoproteins could achieve broader and more durable protection than the targeting of either alone [50,117]. The relatively lower rate of antigenic drift in NA compared to the HA head further supports its inclusion in UIV strategies aimed at reducing the frequency of vaccine reformulation.

### 3.3. Pandemic Preparedness

Influenza pandemics arise when zoonotic influenza A viruses, typically from avian or swine reservoirs, acquire the capacity for sustained human-to-human transmission through antigenic shift, producing viral strains with antigenically novel HA proteins against which the population has little or no pre-existing immunity [11,12,119,120,121,122]. Current pandemic response depends on reactive strain-matched vaccine development, a process requiring months from strain identification to deployment. During this interval, ongoing transmission can continue unchecked, allowing the virus to spread widely and potentially build pandemic momentum before control measures are in place.

A UIV capable of inducing baseline cross-reactive immunity across influenza A groups could fundamentally alter this dynamic. Even partial protection—reducing viral replication, shedding, and disease severity—would slow transmission at the population level, extending the window for strain-specific countermeasure development [11,46]. Mathematical modelling analyses support this: studies projecting the impact of a UIV with moderate cross-protective efficacy estimate meaningful reductions in cumulative pandemic morbidity and mortality relative to reliance on reactive vaccination alone [123,124,125]. However, the magnitude of benefit depends critically on the breadth and durability of the achieved cross-reactive immunity, parameters that remain to be established in large-scale trials.

### 3.4. Manufacturing, Deployment and Economic Considerations

Current SIV production relies primarily on egg-based cultivation, a process constrained by six-month lead times and the risk of egg-adaptive mutations reducing antigenic matching with circulating strains [34,119,126,127]. Several UIV platforms offer meaningful manufacturing advantages over this paradigm. mRNA and recombinant protein vaccines enable rapid, scalable antigen production without live virus handling, while nanoparticle platforms have demonstrated consistent quality characteristics amenable to standardised manufacturing [45,94]. However, not all UIV approaches simplify production—nanoparticle vaccines introduce considerable formulation complexity, and even traditional platforms (whole inactivated and live-attenuated virus) face scalability challenges at global deployment volumes [14,119].

Manufacturing infrastructure for influenza vaccines is disproportionately concentrated in high-income countries. Global scaling of UIV production will require that supply chain inequalities be addressed, particularly for platforms with stringent cold-chain requirements [46,128,129,130,131,132]. Intranasal and thermostable formulations (DeltaFLU and BPL-1357) may offer advantages for resource-limited settings. The ultimate public health impact of any UIV will depend on the achievement of sufficient population coverage, which will require that existing barriers to influenza vaccine uptake be addressed, including cost, access, and public confidence [130,133].

Equally important is the consideration of how a UIV would be integrated into existing influenza immunisation programmes. Given that the majority of current candidates remain in early clinical development, it is unlikely that first-generation UIVs would immediately replace SIVs. Instead, they are more likely to complement existing vaccination strategies by establishing durable immunity against conserved viral antigens while annual SIVs continue to induce strain-specific neutralising antibodies against circulating strains [11,14]. This complementary approach, with periodic administration of UIVs alongside annual SIVs, could establish a baseline level of broad heterosubtypic immunity while maintaining protection against antigenic drift and reducing the impact of seasonal vaccine mismatch, although an optimal implementation strategy remains to be determined [14,28,46].

Successful implementation of a UIV will also greatly depend on public acceptance [43,46]. The prospect of broader protection may improve vaccine acceptance by potentially reducing the need for repeated vaccination [46,49]. However, as many UIV candidates are designed to elicit broad cross-protective immunity by attenuating disease severity rather than providing sterilising immunity, their infection-permissive mechanism may present challenges for public acceptance if vaccine efficacy is perceived solely as the ability to prevent infection [48,49]. Consequently, effective public health communication is essential to ensure the intended benefits of these vaccines are clearly understood by recipients [43,46].

Despite encouraging preclinical findings and favourable results of early-phase clinical trials, no UIV has yet received regulatory approval. This largely reflects the developmental stages of current candidates, as most remain in phase 1 or 2 clinical evaluation [45,56,57,58,59,60,61,62,63,64,65,66,67,68,69,70,71,72,73,74,75,76,77,78,79,80,81]. In addition, regulatory agencies require robust evidence of safety, durability and clinical efficacy across large, diverse populations, which has yet to be demonstrated. Much of this information relies on results from phase 3 clinical trials, which are yet to be completed for UIV candidates [14,115,116].

The economic case for a UIV is supported by modelling studies estimating substantial cost savings through reduced annual antigen reformulation, lower healthcare utilisation as a result of decreased infection rates, and averted pandemic costs. Analyses consistently project that even a moderately effective multi-year UIV would be cost-effective compared with annual seasonal vaccination across multiple healthcare systems [123,124,125]. These projections depend on assumptions regarding vaccine durability, uptake, and breadth of protection that clinical data has not yet validated and should be treated as indicative rather than definitive.

### 3.5. Computational and AI-Assisted Antigen Design

Computational approaches are playing an increasingly central role in UIV candidate design and evaluation. The FluMos-v1 nanoparticle vaccine discussed in Section 2.2 was designed using computational optimisation algorithms to arrange HA antigens from multiple subtypes on a single self-assembling nanoparticle scaffold, maximising the breadth of cross-reactive antibody responses achievable with a single immunogen [67]. Structure-based antigen stabilisation, as employed in the G1mHA and H1ssF candidates, relies on computational modelling of HA conformational dynamics to engineer mutations that lock the stalk domain in its prefusion conformation, ensuring consistent presentation of broadly neutralising epitopes [32,70]. More broadly, machine learning methods are now being applied to predict antigenic evolution trajectories from sequence data, identify conserved epitopes under functional constraint, and model immune-response landscapes across diverse populations [134,135]. These approaches are particularly relevant to UIV development because they offer the potential to anticipate antigenic drift variants before they emerge in circulation and to design immunogens that pre-emptively target predicted future variants. The integration of computational antigen design with high-throughput structural and serological data is likely to accelerate the iterative optimisation of UIV candidates and to inform rational decisions about which epitopes and antigen configurations are most likely to elicit durable, broadly protective immune responses.

### 3.6. Antigenic Drift, Shift, and Viral Persistence

The principal biological challenge for a UIV is that the conserved viral targets it exploits are not invariant. Although the stalk and internal proteins are under strong functional and structural constraint—limiting the rate and extent of mutation relative to the HA head—they can accumulate substitutions under sustained immune pressure, potentially eroding vaccine efficacy over extended timescales [4]. Antigenic shift poses a more acute and less predictable risk: reassortment events can introduce entirely novel HA or NA segments from avian or swine reservoirs, as occurred in the 1957, 1968, and 2009 pandemics [12]. Whether UIV-induced immunity targeting the HA stalk or internal proteins would retain meaningful cross-protection against a pandemic strain with substantial antigenic divergence remains untested.

A related concern is the ecological persistence of influenza A in animal reservoirs—aquatic birds, swine, horses, and poultry—which creates a continuously replenished viral gene pool and makes complete eradication of influenza A through vaccination alone implausible [12,127]. This contrasts with influenza B, which circulates primarily in humans and lacks significant animal reservoirs. The recent extinction of the B/Yamagata lineage demonstrates that influenza lineage elimination can occur in the absence of zoonotic maintenance; a UIV with cross-lineage influenza B protection could theoretically contribute to the elimination of the B/Victoria lineage that still circulates [6,86].

Recent population-level serological studies have provided evidence that cross-lineage antibody responses contributed to B/Yamagata’s extinction. Steventon et al. showed that neutralising immunity was asymmetrically distributed between the two influenza B lineages prior to B/Yamagata’s disappearance and that convergent HA evolution between B/Yamagata and B/Victoria increased B/Yamagata’s susceptibility to cross-neutralisation by Victoria-elicited antibodies [136]. These observations suggest that immunological pressure from cross-reactive antibodies can contribute to lineage elimination and that UIV strategies capable of deliberately eliciting such cross-lineage responses could achieve broader influenza B coverage from a single immunogen.

### 3.7. Immune Imprinting

Immune imprinting represents the most direct immunological barrier to UIV efficacy and warrants specific consideration for this class of vaccines. Prior influenza exposure—through infection or vaccination—establishes immunological memory preferentially directed against the HA head epitopes of the first-encountered strains [111,113,137,138]. Upon subsequent vaccination with a UIV targeting conserved regions, pre-existing memory B cells directed against variable head epitopes are preferentially recalled over naïve or subdominant B cells recognising the intended conserved targets, a phenomenon that can substantially attenuate the intended vaccine response, redirecting humoral immunity away from the cross-protective specificities [139,140,141,142].

Several candidates reviewed here directly address this challenge through distinct mechanisms. Mosaic nanoparticle vaccines (FluMos-v1) present HA antigens from multiple strains on a single particle, forcing B-cell responses towards shared epitopes including the stalk. Clinical data suggests that this approach can elicit stalk-directed antibodies, even in pre-immune individuals with established head-directed memory [45,96]. BPL-1357 circumvents imprinting by using avian influenza subtypes to which humans are immunologically naïve, priming responses uncontaminated by prior head-directed memory. Candidates targeting internal proteins (OVX836, FLU-v and ChAdOx1NP+M1) bypass the HA immunodominance hierarchy entirely, engaging T-cell and non-neutralising antibody responses against NP and M1 that are not subject to the same recall bias. Whether any of these approaches overcomes imprinting across diverse human populations with varied exposure histories remains to be determined in larger trials [138,143].

The consistency of vaccine-induced cross-reactive responses is a further concern. Broadly reactive antibodies can be induced under controlled conditions, but their durability and protective capacity vary substantially between individuals, influenced by age, infection history, and prior vaccination background [128,144,145,146,147]. Establishing whether UIV-induced immunity is sufficiently consistent and long-lived across immunological backgrounds is essential for predicting population-level impact.

Therefore, administering a UIV as part of childhood immunisation could prove strategically advantageous. Children have limited prior influenza exposure, reducing the impact of imprinting on initial UIV responses and potentially allowing the establishment of broader antibody and T-cell repertoires than would be achievable in adults with extensive influenza exposure histories. Additionally, vaccinating children can greatly reduce transmission rates during influenza seasons, benefiting the wider population [111,139,140,141,142].

Although immune imprinting is often regarded as a barrier to the achievement of broad immunity, accumulating evidence suggests that it may not be entirely fixed and could potentially be modified through sequential antigenic exposure [28,36]. Consequently, an important area of future research will be determining whether UIVs can potentially redirect immune responses towards conserved viral epitopes despite pre-existing immune biases. However, the extent to which established imprinting can be reshaped remains uncertain and is likely to vary according to host-related factors such as age, exposure history and immune status [28,36].

## 4. Conclusions

UIVs have significant potential to overcome the fundamental limitations of SIVs, which are heavily constrained by antigenic drift, strain mismatch and the need for annual reformulation [3]. The reviewed platforms have demonstrated immunogenicity across a diverse range of target epitopes with favourable safety profiles [45]. However, results showing early immunogenicity do not necessarily translate into long-term protection. Crucial uncertainties remain regarding the durability of antibody and T-cell responses beyond 12–18 months and the consistency of cross-reactive immunity across populations with differing influenza exposure histories [113,138]. Future research should prioritise robust evaluation of long-term vaccine efficacy in large-scale clinical trials to establish benchmarks for correlates of protection that can be used to interpret trial results.

Additionally, substantial implementation challenges remain. Factors such as manufacturing scalability, cold-chain dependence and equitable global access, particularly in resource-limited settings, are essential, as gaps in coverage will enable continued viral circulation and antigenic evolution [128,129,130,131,132].

Overall, a UIV has the potential to serve as a proactive route for pandemic preparedness and to reduce annual influenza incidence rates. With current advances in vaccine development, a UIV could potentially become available for public distribution and immunisation in the next decade.

## Figures and Tables

**Table 1:** Comparison of reported immune endpoints across UIV candidates by platform category.

Platform	UIV Candidate	Immune Endpoint	References
Recombinant Protein and Peptide	FLU-v	Polyfunctional CD4+ and CD8+ T-cell responses (IFNγ- and granzyme-B-secreting cells)	[62,63]
	FluMos-v1	Robust CD4+ and CD8+ T-cell activation	[67]
	FluMos-v2	Directed IgG responses	[69,72]
Nanoparticle	H1ssF	Stalk-directed antibody responses against group 1 HA stalks	[70]
	OVX836	Anti-NP IgG responses; CD4+ T-cells (IFNγ, IL-2, and TNFα); CD8+ at ≥300 μg	[73,74,75,76,77,78,79]
Nucleic Acid	H1ssF-3928 mRNA-LNP	Directed IgG responses and cross-reactive antibodies against group 1 influenzas	[66]
Whole-Virus	BPL-1357	Strain-specific and cross-reactive HAI titres	[68,79]
Intranasal Live-Attenuated	DeltaFLU	Local immune response; cross-reactive memory T- and B-cells; HAI titres	[58,59]

## Data Availability

No new data were created or analysed in this study. Data sharing is not applicable to this article.
